# A Preliminary Study of Biodegradable Waste as Sorbent Material for Oil-Spill Cleanup

**DOI:** 10.1155/2014/638687

**Published:** 2014-02-11

**Authors:** J. Idris, G. D. Eyu, A. M. Mansor, Z. Ahmad, C. S. Chukwuekezie

**Affiliations:** ^1^Faculty of Mechanical Engineering, Universiti Teknologi Malaysia, Skudai, Johor Bahru 81310, Malaysia; ^2^ISI, Universiti Teknologi Malaysia, Skudai, Johor Bahru 81310, Malaysia

## Abstract

Oil spill constitutes a major source of fresh and seawater pollution as a result of accidental discharge from tankers, marine engines, and underwater pipes. Therefore, the need for cost-effective and environmental friendly sorbent materials for oil spill cleanup cannot be overemphasized. The present work focuses on the preliminary study of empty palm fruit bunch fibre as a promising sorbent material. The morphology of the unmodified empty palm fruit bunch, EPFB fibre, was examined using an optical microcopy, scanning electron microcopy coupled with EDX and X-ray diffraction. The effects of oil volume, fibre weight, and time on oil absorption of EPFB fibre were evaluated with new engine oil from the model oil. The results show that EPFB fibre consists of numerous micro pores, hydrophobic, and partially crystalline and amorphous with approximately 13.5% carbon. The oil absorbency of the fibre increased with the increase in oil volume, immersion time, and fibre weight. However, sorption capacity decreased beyond 3 g in 100 mL. Additionally unmodified EPFB fibre showed optimum oil sorption efficiency of approximately 2.8 g/g within three days of immersion time.

## 1. Introduction

Sorbents are materials with high attractions for oil and repellent for water. Sorbent materials remove oil by two mechanisms. These can either be done by adsorption or absorption. Adsorption involves the adherence of oil to the sorbent material which is dependent upon the viscosity of the oil. The more viscous the oil, the thicker the layer that will adhere to a given material. On the other hand, absorption relies on capillary attraction; oil fills the pores within the material and moves upward (uptake) into the material due to capillary force. Sorbent can be grouped as inorganic minerals and synthetic, organic, and organic (agricultural) products. Agricultural sorbents are cheap, efficient, environmentally friendly, and easy to deploy. However, efficiency is dependent on sorption capacity, density, wettability, retention rate and recyclability [1, 2], and examples are cotton, straws, corn cobs, coconut shells, kenaf, kapok fibres, rice husk, and silkworm cocoon, hay, sawdust, bagasses, gorse, and dried palm fronds [3–6].These materials are oleophilic because of their waxy nature, they become light weight when dried, which improves their buoyancy in water. Straw has been the most widely and probably the most efficient of all these materials. Straw fibres can float on the water surface for a very long period of time to collect oil adequately. It has been reported that straw sorption capacity is higher than commercial synthetic organic material from propylene [7]. Similarly, kapok, rice husk, banana trunk fibre, acetylation of raw cotton, and cotton grass fibre, have been reported to be efficient as oil sorbents [8–13].

Malaysia is the world largest palm oil fruit producer. The palm plantations form an enormous source of fibrous biomass [14]. The empty fruit bunches are normally considered as an agricultural waste and usually left to naturally decompose or dispose at a land fill; this poses environmental challenge. The use of date palm activated carbon as sorbent has been reported [15]. However, very limited study of the use of empty fruit bunch fibre as oil sorbent and factors affecting its absorption capacity has been reported throughout the literature to the best of our knowledge. This present work evaluates the oil sorption capacity of unmodified empty fruit bunch fibre and how its efficiency is influenced by oil volume, time, and fibre weight for engine oil cleanup.

## 2. Materials and Methods

### 2.1. Materials

The empty fruit waste was obtained from the palm oil factory in the area of Jengka, Pahang State, Malaysia, Sea water was collected from the Danga bay beach in Johor Bahru, Malaysia, and was used at room temperature. New engine oil with the characteristics shown in Table 1 was used for the study.

### 2.2. Preparation of EPFB

**Figure 1:**
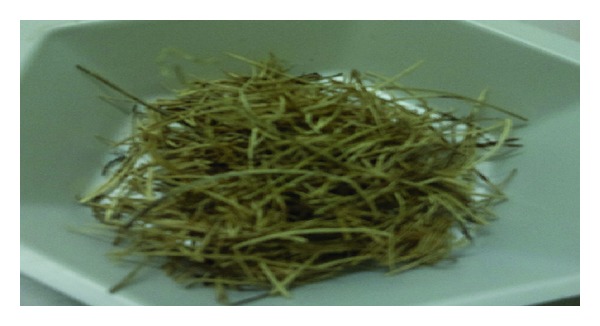
EPFB fibre.

The empty palm fruit bunch was chopped into smaller pieces and deoiled by soaking in hot deionized water with detergent for 24 hr. The sample was rinsed several times with distilled water to remove all debris and then air-dried at the ambient temperatures. The air-dried fibre was ground using electric blender and sieved to obtain a desired fibre size of 106 *μ*m as shown in Figure 1.

### 2.3. Absorbent Characterizations

The EPFB was observed by Philips XL40 scanning electron microscopy coupled with EDX model Philips XL 40 PW 6822/10 at an accelerating voltage of 15 kV. Before SEM examination, the samples were gold-coated with a thin layer of approximately 30 nm sputter coater with cool sputter coater (bio-rad) Edwards E2 M5. D5000 Siemens X-ray diffraction (XRD) machine was also used to examine the crystalline patterns of the fiber. The diffractograms were in the range of 2*θ* from 10° to 60° with 0.050° steps at room temperature.

### 2.4. Oil Sorption Capacity

A 500 mL sample of sea water was placed in a 1 L glass beaker, 40 mL oil was added to the beaker, and the mixture was shaken. The dried fibre sample (1 g) was weighed in a steel mesh and then poured into the beaker containing the oil mixture in sea water. Shaking time was approximately 15 minutes at 105 cycles/min. Thereafter, the wetted fibre was weighed after being drained for 5 min in the oven [16]. The test was repeated three times in order to obtain the average values. The oil sorption of the sample was evaluated by weighing the samples before and after the absorption and determined by the formula:

(1) $$\begin{array}{c} \text{Oil}\;\text{sorption}\;\text{capacity}\;\left(\text{OSC}\right):\frac{\left[\text{ST}-\text{SC}-\text{SA}\right]}{\text{SA}}. \end{array}$$

SA is dry weight of sorbent (g), ST is total weight of oil (g), water, and dry sorbent, and SC is weight of water (g).

The experiment was repeated with varied oil volume, fibre's weight, and sorbent immersion durations.

### 2.5. Evaluation of Cyclic Sorption/Desorption Characteristic

The fibre was removed from the water/oil mixture systems with the aid of the mesh screen. The oil was then drained by squeezing the absorbed oil from the test cells manually after the amount of absorbed oil was determined. The weight of the test cells and the squeezed oil were measured in each cycle. The process was repeated until the sorption capacity result was less than 50% from the first cycle [17].

## 3. Results and Discussion

### 3.1. Characterization

#### 3.1.1. SEM-EDX Study

**Figure 2:**
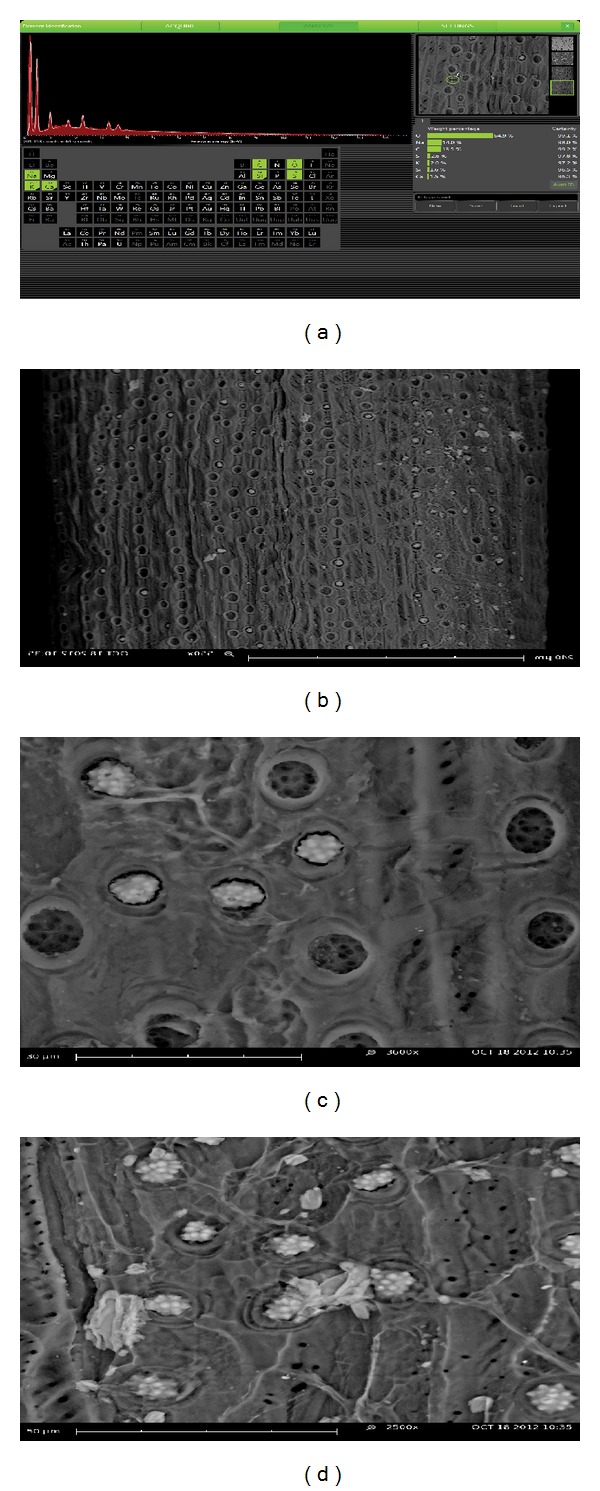
(a) EDX micrograph and (b–d) SEM micrographs of different magnifications.

Figures 2(a) and 2(b)–2(d) show the micrographs of EDX and SEM analyses, respectively; it is evident that EPFB fibre comprises numerous elements such as oxygen, sodium, carbon, sulphur, potassium, silicon and calcium with an approximated percentage quantity of 64.5, 14, 13.5, 2.6, 2, 1.6, and 1.5, respectively. The SEM micrographs revealed that EPFB fibre contained numerous pores, which can transport and hold oil. The pores vary in sizes and are distributed over the silica craters present. The spiky whitish materials are silica bodies, and underneath are perforations, which aid oil sorption [18].

#### 3.1.2. XRD Analysis

**Figure 3:**
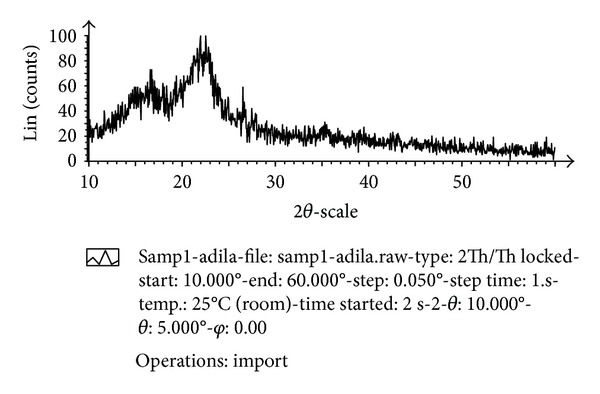
XRD patterns of EPFB fibre.

The XRD pattern of the EPFB is shown in Figure 3. It is observed that two broad peaks appear at 16° and 23° in the crystalline patterns which typify *α*-cellulose XRD pattern [19]. The diffraction patterns at 2*θ* positions, 16.5°, 22°, and 27° show the presence of microcrystalline cellulose I and lignin as reported in [20].

### 3.2. Variation of Liquid Volume (Oil)

The effect of oil volume on the oil sorption capacity was determined as shown in Table 2. The sorption capacity was evaluated as stated in Section 2.4.

**Figure 4:**
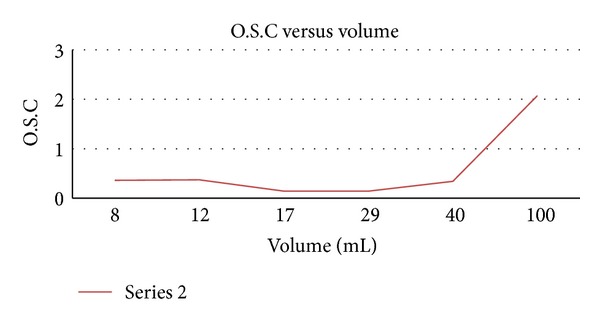
Oil sorption capacity versus liquid volume.

From Figure 4, it is obvious that increment in oil quantity affects the absorption rate of the fibre. The sorption capacity was below 0.5 g/g 5 g/g for engine oil volumes ranging from 8 to 55 mL. However, as the volume of oil was increased beyond 40 mL, the rate of absorption increased significantly and reaches 2.07 g/g of engine oil at 100 mL.

### 3.3. Variation of Fibre Weight

The data of the effect of sorbent weight on sorption capacity is shown in Table 3.

**Figure 5:**
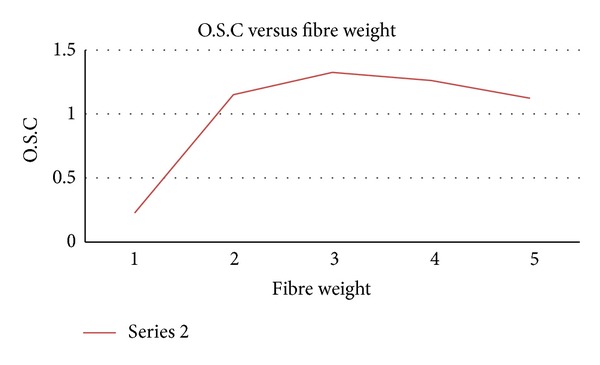
Oil sorption capacity versus fibre weight.

The effect of fibre weight on the oil sorption capacity is illustrated in Figure 5. It was observed that oil sorption increased with increasing fibre weight. However, a further increasing in fibre weight above 3 g does not favour the rate of oil sorption capacity. The efficiency of sorption capacity depends on the fibre weight. The result showed that the sorption capacity of EPFB fibre decreased significantly from 1.33 g/g to 1.12 g/g 12 g/g 12 g/g at fibre weighed 3 g and 5 g, respectively. This trend is similar to the effect of packing density on oil absorption capacity as reported in [20].

### 3.4. Variation of Time Consumed

**Figure 6:**
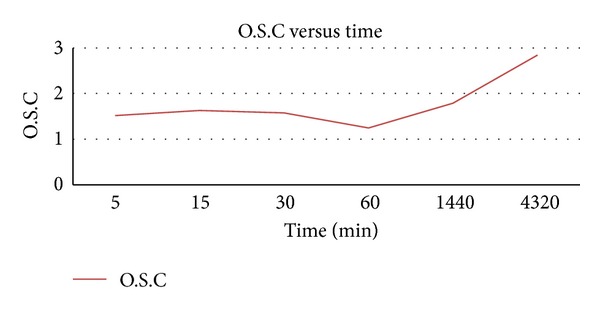
Oil sorption capacity versus time consumption.

Figure 6 shows the relationship between time and absorption capacity of EPBF. It is evident from the figure that there was no significant change in oil sorption within the first 60 minutes. However, there was a considerable increasing of oil sorption from 1.27 g/g to 2.8 g/g after 1 hr and 4320 hr, respectively.

## 4. Conclusions

Empty palm fruit bunches are generally disposed as wastes. The fibres are light weight, which promotes its buoyancy. From the preliminary study of this fibre, it can be concluded that EPFB can be used for oil cleanup. The use of the empty palm fruit bunch is environmentally friendly, sustainable, and economical. The residue of the oil-based sorbents of empty palm fruit bunch waste at the end of its life cycle can be used as high-energy fuel. However, the need for further research to investigate other characteristics besides the one presented throughout this study is essential.

## Figures and Tables

**Table 1:** Properties of liquid used in experiment.

Liquid	Density @ 20°C	Viscosity @ 40°C	Grade
Engine oil	0.866	65 m^2^/s	5W-30

**Table 2:** Data of the variation of liquid volume test.

*V*/g	SC	SA	ST	O.S.C
100	0.8312	1.0208	3.9662	2.0711
40	0.8213	1.2842	2.5457	0.3428
29	0.7990	1.1635	2.1330	0.1465
17	0.8265	1.2434	2.2526	0.1469
12	0.7794	1.2248	2.4598	0.3719
8	0.7952	1.2663	2.5194	0.3616

**Table 3:** Data of the variation of fibre weight test.

Wt.	SC	SA	ST	O.S.C
(1)	0.8140	1.2463	2.3451	0.2285
(2)	0.7954	2.0216	5.1449	1.1515
(3)	0.8072	3.1163	8.0562	1.3262
(4)	0.7987	4.2370	10.3800	1.2613
(5)	0.7924	5.0658	11.5517	1.1239
